# Frequency-domain stability analysis of mixed traffic flow considering communication degradation and human driving heterogeneity

**DOI:** 10.1371/journal.pone.0352664

**Published:** 2026-07-01

**Authors:** Haotian Han, Fangyuan Li, Zhihu Hao, Long Qi, Zihan Wei, Changli Zhao

**Affiliations:** 1 School of Automotive Engineering, Shandong Jiaotong University, Jinan, China; 2 Industry Policy Department, China Road Transport Association, Beijing, China; Beijing Institute of Technology, CHINA

## Abstract

Mixed traffic containing connected automated vehicles (CAVs) and human-driven vehicles (HDVs) is sensitive to loss of cooperative information and heterogeneous driver responses. This study develops a probability-weighted full-frequency transfer-function framework to evaluate longitudinal string stability under communication degradation. CAVs are represented by cooperative adaptive cruise control (CACC) when cooperative information is available and by adaptive cruise control (ACC) when such information is unavailable; HDV responses are represented by the intelligent driver model (IDM), optimal velocity model (OVM), and risk-sensitive model (RSM). The framework preserves frequency-dependent disturbance responses and interprets the conventional algebraic condition as a low-frequency approximation. Under the adopted single-lane, small-perturbation assumptions, passive CACC-to-ACC degradation raised the global critical CAV penetration threshold from about 87.3% in an ideal connected-upgrade benchmark to about 96.0% in the degraded baseline. HDV heterogeneity mainly compressed local stable-speed regions rather than changing the global threshold, and this pattern persisted under 100 Latin Hypercube samples with ±10% HDV parameter perturbations. A 5% functional communication-reliability loss increased the critical threshold to about 99.7% and compressed the stable domain; an equivalent response delay reduced the stable-area ratio even when the global threshold changed only slightly at 0.20 s. An NGSIM US101 trajectory replay supported the IDM-based HDV component, with median speed and spacing root mean square errors of 0.8456 m/s and 3.5078 m. These findings indicate that mixed-flow stability depends not only on CAV penetration but also on cooperative-information availability and HDV response heterogeneity, while the full speed-penetration stability map remains a model-based prediction.

## 1. Introduction

Microscopic instability in car-following behavior is a core factor leading to typical traffic oscillations and phantom jams [1–5]. CAVs have been proposed as a potential means to mitigate these oscillations through vehicle-to-everything (V2X) cooperative platooning [6–7]. However, a prolonged transitional stage characterized by mixed traffic flows, comprising CAVs, communication-degraded automated vehicles (AVs), and HDVs, is inevitable [8–9]. Assessing the platoon stability of such mixed flows remains a core challenge in facilitating the smooth transition toward full automation.

Recent CAV and V2X research further expands this transition context. Reinforcement-learning-based highway autonomous vehicle control has been reviewed as an important control direction [10]. The alignment between platoon control approaches and clustering strategies has also been comprehensively examined [11]. Reinforcement-learning-based end-to-end decision-making has been investigated for autonomous driving on roads with consecutive sharp turns [12]. In the communication and estimation dimensions, optical communication based V2V has been explored for vehicle platooning [13], and an interacting-multiple-model-based ETUKF has been used for efficient state estimation of connected vehicles with V2V communication [14]. These studies broaden the technical background of CAV/V2X deployment, while the present study focuses on the more specific stability problem caused by communication degradation and HDV heterogeneity.

Traditionally, algebraic discrimination frameworks have been widely employed to delineate macroscopic stability boundaries because of their concise analytical structures [15–17]. However, these methods typically invoke a homogenization assumption, uniformly averaging microscopic car-following stiffness and obscuring critical physical differences among heterogeneous individuals [18–19]. This evaluation bias may underestimate actual safety margins and cause boundary misalignments of up to 11% [20]. Consequently, an important research gap remains: full-frequency analysis is needed to delineate where the local equivalence of algebraic discriminants fails across broader penetration rates.

Furthermore, mixed traffic flow stability relies profoundly on the multidimensional physical heterogeneity of microscopic entities. While existing studies have predominantly focused on isolated factors such as CAV penetration rates [21–22], real-world dynamics involve complex degradation mechanisms. Specifically, when CAVs follow non-connected HDVs, their cooperative capacity degenerates from cooperative adaptive cruise control (CACC) to standard adaptive cruise control (ACC), inducing local damping attenuation that can trigger nonlinear traffic distortions [8,23]. Coupled with this is the inherent multidimensional heterogeneity of human drivers, characterized by mechanical sluggishness, perception errors, and risk-averse operational fluctuations [24–25]. Current research still lacks a unified frequency-domain framework to elucidate how the coupling of ACC communication degradation and human cognitive/mechanical biases affects macroscopic stability boundaries.

To address these theoretical blind spots and mechanistic gaps, this paper constructs a stochastic probability space based on the unified linearization of nonlinear car-following models, thereby extracting frequency-domain response functions for CACC, ACC, and three heterogeneous HDV types. This framework establishes the theoretical relationship between frequency-domain strong conditions and algebraic weak conditions, and supports a model-based comparison between macroscopic stability boundaries and microscopic time-domain dynamics across different speed phases.

This paper treats random CAV distribution as a non-organized early-stage setting rather than a universal deployment strategy. AVs are defined only as the passive degraded state of CAVs operating in ACC mode after cooperative-information loss. A delay-free baseline isolates communication-state degradation and HDV heterogeneity, while equivalent delay, HDV-side parameter uncertainty, functional communication reliability loss, and NGSIM US101 IDM replay define robustness and empirical boundaries. The US101 replay supports the IDM-based HDV component; the full CAV-ACC-HDV stability boundary remains a frequency-domain model prediction.

The main contributions of this study are as follows:

1. It uses second-order Taylor expansion to show that the algebraic discriminant is a long-wave, low-frequency equivalent of the frequency-domain discriminant, thereby clarifying where boundary evaluation misalignment can occur.
2. It analyzes how communication degradation and heterogeneous human driving responses jointly affect stability across different speed phases, with the interpretation kept within the adopted model-based assumptions.
3. By establishing an ideal connected-upgrade benchmark, it quantifies the stability benefit of maintaining cooperative information availability without treating this benchmark as a direct deployment strategy.
4. It extends the original numerical and time-domain analyses with HDV-side parameter uncertainty, functional communication reliability loss, equivalent response-delay sensitivity, and US101-based IDM trajectory replay.

## 2. Selection of car-following models and stability evaluation of homogeneous platoons

### 2.1 Main modeling assumptions and scope

The modeling assumptions used to isolate communication degradation and HDV heterogeneity are:

(1) System dynamics are strictly confined to longitudinal single-lane car-following, neglecting lateral maneuvers and resultant disturbances.
(2) CAV spatial distribution is completely random; the system does not actively assemble dedicated platoons. Vehicle composition is defined via probability proportions rather than deterministic structures, and CAVs following HDVs passively degenerate into AVs. This baseline degradation mechanism is further extended in Section 4.6 by introducing a functional cooperative-information unavailability probability.
(3) The main analytical framework first adopts a delay-free baseline to isolate the effects of communication-state degradation and HDV heterogeneity. To examine the influence of this simplification, an equivalent response-delay sensitivity analysis is further added in Section 4.7.
(4) AVs are not defined as an independent category but are strictly treated as a passive degraded state of CAVs experiencing V2X communication breakage.

These assumptions define a baseline analytical setting, not a complete mixed-traffic representation. The single-lane model isolates longitudinal string stability; lane changing, merging, diverging, and active platoon organization are left for future work. Random distribution represents an early-stage mixed-traffic environment without dedicated platoon formation.

### 2.2 CAV car-following model and its communication degradation model

#### 2.2.1 Ideal communication state: CACC model.

Integrating advanced perception and execution modules, CAVs possess automated driving capabilities and V2X communication functionalities [26]. The ideal cooperative state is represented by the CACC model [27]:


$$\begin{array}{c} v_{n}(t+\Delta t)=v_{n}(t)+k_{p}e_{n}(t)+k_{d}\dot{e_{n}}(t) \end{array}$$ (1)

$$\begin{array}{c} e_{n}(t)=\Delta x_{n}(t)-\tau_{c}\nu_{n}(t) \end{array}$$ (2)

where $k_{p}$ and $k_{d}$ denote gains adjusting the time gap, with recommended experimental values of $k_{p}=\text{0}.\text{4}\text{5}s^{-\text{1}}$ and $k_{d}=\text{0}.\text{2}\text{5}s^{-\text{1}}$ [27]. Given the random spatial distribution without dedicated platooning, the car-following time gap is set to $\tau_{c}=\text{0}.\text{6}\text{s}$ [28,29]. Applying a first-order Taylor expansion yields the acceleration:


$$\begin{array}{c} {\text{a}}_{\text{n}}(\text{t})=\frac{{\text{k}}_{\text{p}}(\Delta {\text{x}}_{\text{n}}(\text{t})-\tau_{\text{c}}\nu_{\text{n}}(\text{t}))+{\text{k}}_{\text{d}}\Delta \nu_{\text{n}}(\text{t})}{{\text{k}}_{\text{d}}{\text{t}}_{\text{c}}+\Delta \text{t}}, \end{array}$$ (3)

where Δt is the time step, set to 0.01s.

#### 2.2.2 Communication degradation state: ACC model.

When a CAV follows a vehicle lacking V2X communication capabilities, its cooperative capability is restricted. An ACC model calibrated with experimental data is adopted as its degraded form [27]:


$$\begin{array}{c} a_{n}(t)=k_{\text{1}}(\Delta x_{n}(t)-\tau_{a}\nu_{n}(t))+k_{\text{2}}\Delta \nu_{n}(t), \end{array}$$ (4)

where $k_{\text{1}}=\text{0}.\text{23}s^{-\text{1}}$ and $k_{\text{2}}=\text{0}.\text{07}s^{-\text{1}}$ represent position and speed error gains, respectively, with a time gap of $\tau_{\text{a}}=\text{1}.\text{5s}$ [27,29].

### 2.3 Heterogeneous HDV car-following models

To represent HDV perception and risk-preference differences, this study uses three established car-following models as representative mechanisms rather than new models. IDM represents safety-distance and relative-speed-coupled response; OVM represents spacing-driven response without explicit relative-speed feedback; RSM represents risk-sensitive stochastic response. This library allows the frequency-domain framework to test how HDV mechanisms reshape mixed-flow stability boundaries.

#### 2.3.1 Intelligent driver model (IDM).

The IDM was developed by Treiber et al. (2000), and its governing equation is given by:


$$\begin{array}{c} a_{n}(t)=A\left[\text{1}-{\left(\frac{\nu_{n}(t)}{V}\right)}^{\text{4}}-{\left(\frac{s_{\text{0}}+\tau_{h}\nu_{n}(t)+\frac{\nu_{n}(t)\Delta \nu_{n}(t)}{\text{2}\sqrt{AB}}}{\Delta x_{n}(t)}\right)}^{\text{2}}\right], \end{array}$$ (5)

where V=33.3m/s is the free-flow speed; $s_{\text{0}}=\text{2}\text{m}$ is the minimum jam distance; $A=\text{1}\text{m}/{\text{s}}^{\text{2}}$ and $B=\text{2}\text{m}/{\text{s}}^{\text{2}}$ denote maximum acceleration and desired deceleration; and $\tau_{h}=\text{1}.\text{6}\text{s}$ is the safe time headway [30,31]. Structurally, the IDM relies on target speed and safe distance, exhibiting strong nonlinear coupling, high gain, and robust damping upon linearization.

#### 2.3.2 Optimal velocity model (OVM).

The OVM was proposed by Bando [32], and its model equation is:


$$\begin{array}{c} a_{n}(t)=\kappa [V(h_{n}(t))-\nu_{n}(t)], \end{array}$$ (6)

where κ is the sensitivity coefficient, and V(·) is the optimal velocity function [33]:


$$\begin{array}{c} V(h_{n}(t))=\nu_{\text{0}}\left[\text{1}-\text{exp}(-\frac{\lambda }{\nu_{o}}(h_{n}(t)-d)\right], \end{array}$$ (7)

with recommended parameters $K=\text{0}.\text{7}\text{0}\text{0}s^{-\text{1}}$, $\lambda =\text{0}.\text{9}\text{9}\text{9}s^{-\text{1}}$, and d=1.62m. Lacking explicit relative speed feedback, the OVM operates as a flexible structure exhibiting moderate gain and high spacing sensitivity.

#### 2.3.3 Risk-sensitive model (RSM).

The RSM was proposed by Hamdar [34]. To model perception errors, the driver’s acceleration disturbance is represented by a Wiener process:


$$\begin{array}{c} a(t)=a^{*}(t)+\sigma_{a}(t)y(t) \end{array}$$ (8)

$$\begin{array}{c} y(t)=y(t-\Delta t)e^{-\Delta t/\tau }+\sqrt{\text{2}\text{4}\Delta t/\tau }(z-\text{0}.\text{5}) \end{array}$$ (9)

where $a^{*}(t)$ is the optimal acceleration, $\sigma_{a}(t)$ is the fluctuation amplitude, and y(t) is a standard Wiener process. With z uniformly distributed over [0,1], (z−0.5), and τ denoting the correlation time. Assuming drivers maximize utility under risk aversion, the total utility is defined as:


$$\begin{array}{c} U_{PT}(a|s,\Delta v,v)=a-w_{c}\Phi (z(a|s,\Delta v,v)) \end{array}$$ (10)

where Φ(z) is the density function of the Gaussian distribution, $z(a|s,\Delta \nu ,\nu )=\frac{\Delta \nu +\frac{\text{1}}{\text{2}}a\tau -\frac{s}{\text{2}}}{\alpha \nu }$, and:


$$\begin{array}{c} \tau (s,\Delta v)=\left\{ \begin{array}{cccc} & \frac{s}{\Delta v}\; & & \Delta v>\frac{s}{\tau_{max}} \end{array}\right.\; \end{array}$$

Maximizing Eq. (10) yields the optimal acceleration. After linearization, the RSM reflects risk-averse speed sensitivity and stochastic execution.

### 2.4 Local stability evaluation of homogeneous platoons and model establishment

Prior to evaluating multi-class mixed traffic flows, this study utilizes the algebraic discrimination method [35] to assess the local asymptotic stability of the homogeneous platoons:


$$\begin{array}{c} F=\frac{\text{1}}{\text{2}}(f_{\nu }{)}^{\text{2}}-f_{\Delta \nu }f_{\nu }-f_{\Delta x}+\frac{f_{\nu }}{f_{\Delta x}}\left(\overset{\Delta x}{\underset{n}{\xi }}-\overset{\nu }{\underset{n}{\xi }}\right)>\text{0}, \end{array}$$ (11)

where $\xi \Delta n_{x}=\xi v=\text{0}$, and *n* denotes the vehicle type, which can be omitted unless necessary. The expansion coefficients are defined as:


$$\begin{array}{c} \left\{ \begin{array}{c} @lf_{h}=\frac{\partial f\left(h_{n},\Delta v_{n},v_{n}\right)}{\partial h_{n}}{|}_{\left({\text{h}}^{*},\text{0},V(h^{*})\right)}\\ f_{\Delta v}=\frac{\partial f\left(h_{n},\Delta v_{n},v_{n}\right)}{\partial \Delta v_{n}}{|}_{\left(h^{*},\text{0},V(h^{*})\right)}\\ f_{v}=\frac{\partial f\left(h_{n},\Delta v_{n},v_{n}\right)}{\partial h_{v}}{|}_{\left(h^{*},\text{0},V(h^{*})\right)}. \end{array}\right.\; \end{array}$$ (12)

The partial derivatives of the five models are evaluated at equilibrium and substituted into the discriminant to obtain stability indicators over 0–30 m/s.

**Fig 1:**
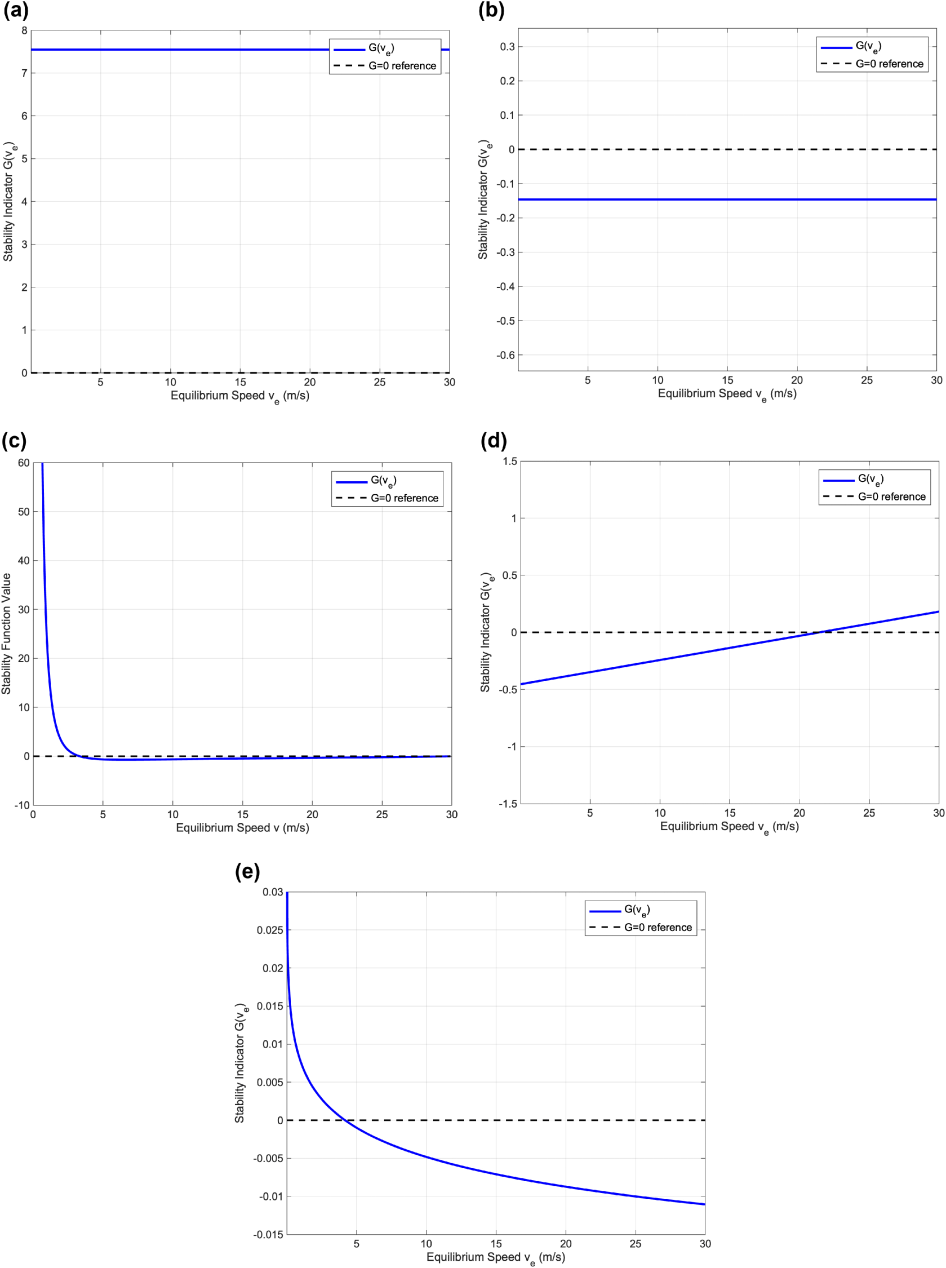
Stability analysis of homogeneous traffic flows.

Figure 1 shows that CACC remains stable over the examined speeds, whereas ACC is unstable under the same local criterion, supporting the treatment of AVs as passive CAV degradation states when cooperative information is unavailable. Among HDV models, IDM and RSM are stable at low speeds and unstable at high speeds, while OVM shows low-speed instability because of its spacing-sensitive formulation and lack of explicit relative-speed feedback.

IDM is used as the baseline HDV component in Sections 4.1–4.3 because it is comparatively stable and widely used. The IDM-OVM-RSM library is then used in Section 4.4 to test how HDV response mechanisms reshape local frequency-domain stability boundaries.

The empirical plausibility of the IDM baseline is examined through US101 trajectory replay in Section 4.8. This replay is limited to the IDM-based HDV component because the dataset lacks CAV/CACC labels and communication-degradation states.

## 3. Modeling of mixed traffic flow and frequency-domain stability discrimination criteria

### 3.1 stochastic probability distribution model of mixed traffic flow

Assuming a spatial distribution of vehicles based on independent Bernoulli trials, the communication topology within the mixed traffic flow is strictly governed by the preceding-following relationship. With a basic CAV penetration rate of p and an HDV proportion of 1−p, the absolute probability of the undegraded cooperative state is $P_{CACC}=p^{\text{2}}$, occurring solely when a CAV follows another CAV. Conversely, communication degradation emerges when a CAV is forced to follow a non-connected HDV, yielding $P_{ACC}={p-p}^{\text{2}}$. The HDV state remains independent of preceding communication conditions, thus $P_{HDV}=\text{1}-p$.

To capture real-world multidimensional heterogeneity, we further subdivide the human-driven group by introducing internal composition proportion factors $q_{\text{1}}$, $q_{\text{2}}$, and $q_{\text{3}}$ ($\sum q_{i}=\text{1}$) for the IDM (baseline), OVM (flexible), and RSM (risk-sensitive) units, respectively. Consequently, the global mixed traffic flow probability space is defined by five mutually exclusive and collectively exhaustive dynamic states: $P_{CACC}=p^{\text{2}},P_{ACC}=p(\text{1}-p),P_{IDM}=(\text{1}-p)q_{\text{1}},P_{OVM}=(\text{1}-p)q_{\text{2}}$, and $P_{RSM}=(\text{1}-p)q_{\text{3}}$.

### 3.2 Linearization of car-following models and derivation of transfer functions

To investigate the frequency-domain propagation characteristics of small perturbations, the nonlinear models from Section 2 are linearized. Assuming the *n*-th vehicle’s acceleration is $a_{n}(t)=f^{n}(v_{n}(t),h_{n}(t),\Delta v_{n}(t))$ and the system operates at an equilibrium state $\left(v_{e},h_{e},\text{0}\right)$, the perturbation variables are defined as $\Delta u_{n}(t)=u_{n-\text{1}}(t)-u_{n}(t)$, and $\Delta u_{n}(t)=u_{n-\text{1}}(t)-u_{n}(t)$. A first-order Taylor expansion yields:


$$\begin{array}{c} {\dot{u}}_{n}(t)=\overset{n}{\underset{v}{f}}u_{n}(t)+\overset{n}{\underset{\Delta v}{f}}\Delta u_{n}(t)+\overset{n}{\underset{h}{f}}y_{n}(t) \end{array}$$ (13)

where $\overset{n}{\underset{v}{f}}$, $\overset{n}{\underset{\Delta v}{f}}$, and $\overset{n}{\underset{h}{f}}\;$ are the steady-state partial derivatives constituting the system’s stiffness and damping core.

Applying the Laplace transform to Eq. (13), the error transfer function G(s) from the preceding vehicle n−1 to the following vehicle n is:


$$\begin{array}{c} G_{n}(s)=\frac{U_{n}(s)}{U_{n-\text{1}}(s)}=\frac{\overset{n}{\underset{\Delta v}{f}}s+\overset{n}{\underset{h}{f}}}{s^{\text{2}}+(\overset{n}{\underset{\Delta v}{f}}-\overset{n}{\underset{v}{f}})s+\overset{n}{\underset{h}{f}}} \end{array}$$ (14)

Substituting s=jω (ω≥0 is the angular frequency) maps this to the frequency-domain response function:


$$\begin{array}{c} G_{n}(j\omega )=\frac{\overset{n}{\underset{h}{f}}+\overset{n}{\underset{\Delta v}{f}}j\omega }{(\overset{n}{\underset{h}{f}}-\omega^{\text{2}})+(\overset{n}{\underset{\Delta v}{f}}-\overset{n}{\underset{v}{f}})j\omega } \end{array}$$ (15)

Here, the numerator dictates perturbation gain, while the denominator term $\left(\overset{n}{\underset{\Delta v}{f}}-\overset{n}{\underset{v}{f}}\right)$ defines the core physical damping for absorbing high-frequency oscillations.

### 3.3 Unified frequency-domain stability discrimination criterion for mixed traffic flow

Strict string stability necessitates that upstream-propagating perturbations converge macroscopically without amplification at any frequency, mathematically requiring |G(jω)|≤1,∀ω≥0. Extending this to complex mixed traffic flows governed by the established stochastic probability space, the unified frequency-domain composite stability criterion is formulated as:


$$\begin{array}{c} \prod_{i}\;|G_{i}(j\omega ){|}^{P_{i}}\leq \text{1},\forall \omega \geq \text{0} \end{array}$$ (16)

Substituting the established five microscopic states into this generalized framework yields the core constraint equation used for system stability evaluation in this study:


$$\begin{array}{c} |G_{CACC}(j\omega ){|}^{p^{\text{2}}}\cdot |G_{ACC}(j\omega ){|}^{p(\text{1}-p)}\cdot |G_{IDM}(j\omega ){|}^{(\text{1}-p)q_{\text{1}}}\cdot |G_{OVM}(j\omega ){|}^{(\text{1}-p)q_{\text{2}}}\cdot |G_{RSM}(j\omega ){|}^{(\text{1}-p)q_{\text{3}}}\leq \text{1},\forall \omega \geq \text{0} \end{array}$$ (17)

Physically, the framework treats mixed-flow string stability as a probability-weighted attenuation product and tests whether stable vehicle states offset amplification from communication-degraded or heterogeneous HDV responses across the frequency spectrum.

Nominal stability boundaries are first evaluated, then combined with HDV-side parameter uncertainty in Section 4.5 to test dependence on a single parameter set.

### 3.4 Theoretical discrimination between algebraic and frequency-domain discriminants

This section relates the proposed frequency-domain framework to traditional algebraic discriminants.

#### 3.4.1 General algebraic stability condition for mixed traffic flow.

The classic algebraic discrimination framework typically evaluates long-wave asymptotic stability via the following condition:


$$\begin{array}{c} \sum_{n}\;\left[\frac{{\left(\overset{n}{\underset{\nu }{f}}\right)}^{\text{2}}}{\text{2}}-\overset{n}{\underset{\nu }{f}}\overset{n}{\underset{\Delta \nu }{f}}-\overset{n}{\underset{\Delta x}{f}}\right]{\left[\prod_{m\neq n}\;\overset{m}{\underset{\Delta x}{f}}\right]}^{\text{2}}>\text{0} \end{array}$$ (18)

where m and n denote discrete vehicle indices, and $f_{\nu }$, $f_{\Delta \nu }$, $f_{\Delta x}$ represent the respective steady-state partial derivatives.

#### 3.4.2 Derivation of the algebraic discriminant for heterogeneous traffic flow.

Substituting the absolute distribution probabilities ($p^{\text{2}},p(\text{1}-p),\text{and }\text{1}-p$) for the CACC, ACC, and baseline HDV (IDM) modes into Eq. (18), and consolidating the continuous product terms yields:


$$\begin{array}{cc} & |N{|p}^{\text{2}}\left[\frac{\text{1}}{\text{2}}{\left(\overset{\text{CACC}}{\underset{\nu }{f}}\right)}^{\text{2}}-\overset{\text{CACC}}{\underset{\Delta \nu }{f}}\overset{\text{CACC}}{\underset{\nu }{f}}-\overset{\text{CACC}}{\underset{\Delta x}{f}}\right]{\left[{\left(\overset{\text{HDV}}{\underset{\Delta x}{f}}\right)}^{N_{\text{HDV}}}{\left(\overset{\text{ACC}}{\underset{\Delta x}{f}}\right)}^{N_{\text{ACC}}}{\left(\overset{\text{CACC}}{\underset{\Delta x}{f}}\right)}^{N_{\text{CACC}}-\text{1}}\right]}^{\text{2}}\\ & +|N|p(\text{1}-p)\left[\frac{\text{1}}{\text{2}}{\left(\overset{\text{ACC}}{\underset{\nu }{f}}\right)}^{\text{2}}-\overset{\text{ACC}}{\underset{\Delta \nu }{f}}\overset{\text{ACC}}{\underset{\nu }{f}}-\overset{\text{ACC}}{\underset{\Delta x}{f}}\right]{\left[{\left(\overset{\text{HDV}}{\underset{\Delta x}{f}}\right)}^{N_{\text{HDV}}}{\left(\overset{\text{ACC}}{\underset{\Delta x}{f}}\right)}^{N_{\text{ACC}}-\text{1}}{\left(\overset{\text{CACC}}{\underset{\Delta x}{f}}\right)}^{N_{\text{CACC}}}\right]}^{\text{2}}\\ & +|N|(\text{1}-p)\left[\frac{\text{1}}{\text{2}}{\left(\overset{\text{HDV}}{\underset{\nu }{f}}\right)}^{\text{2}}-\overset{\text{HDV}}{\underset{\Delta \nu }{f}}\overset{\text{HDV}}{\underset{\nu }{f}}-\overset{\text{HDV}}{\underset{\Delta x}{f}}\right]{\left[{\left(\overset{\text{HDV}}{\underset{\Delta x}{f}}\right)}^{N_{\text{HDV}}-\text{1}}{\left(\overset{\text{ACC}}{\underset{\Delta x}{f}}\right)}^{N_{\text{ACC}}}{\left(\overset{\text{CACC}}{\underset{\Delta x}{f}}\right)}^{N_{\text{CACC}}}\right]}^{\text{2}}>\text{0} \end{array}$$ (19)

where $|N|=N_{\text{HDV}}+N_{\text{ACC}}+N_{\text{CACC}}$. Given |N|>0 and $f_{\Delta x}>\text{0}$ for all vehicle types, extracting the global product common factor simplifies the inequality to:


$$\begin{array}{c} p^{\text{2}}G_{\text{CACC}}+p(\text{1}-p)G_{\text{ACC}}+(\text{1}-p)G_{\text{HDV}}>\text{0} \end{array}$$ (20)

where G represents the macroscopic algebraic stability discrimination value of the mixed traffic flow, and the algebraic attenuation cores for each sub-term are defined as:


$$\begin{array}{c} \left\{ \begin{array}{cccc} & G_{\text{CACC}}\; & & =\frac{\frac{\text{1}}{\text{2}}{\left(\overset{\text{CACC}}{\underset{\nu }{f}}\right)}^{\text{2}}-\overset{\text{CACC}}{\underset{\Delta \nu }{f}}\overset{\text{CACC}}{\underset{\nu }{f}}-\overset{\text{CACC}}{\underset{\Delta x}{f}}}{{\left(\overset{\text{CACC}}{\underset{\Delta x}{f}}\right)}^{\text{2}}}=\frac{F_{\text{CACC}}}{{\left(\overset{\text{CACC}}{\underset{\Delta x}{f}}\right)}^{\text{2}}}\\ & G_{\text{ACC}}\; & & =\frac{\frac{\text{1}}{\text{2}}{\left(\overset{\text{ACC}}{\underset{\nu }{f}}\right)}^{\text{2}}-\overset{\text{ACC}}{\underset{\Delta \nu }{f}}\overset{\text{ACC}}{\underset{\nu }{f}}-\overset{\text{ACC}}{\underset{\Delta x}{f}}}{{\left(\overset{\text{ACC}}{\underset{\Delta x}{f}}\right)}^{\text{2}}}=\frac{F_{\text{ACC}}}{{\left(\overset{\text{ACC}}{\underset{\Delta x}{f}}\right)}^{\text{2}}}\\ & G_{\text{HDV}}\; & & =\frac{\frac{\text{1}}{\text{2}}{\left(\overset{\text{HDV}}{\underset{\nu }{f}}\right)}^{\text{2}}-\overset{\text{HDV}}{\underset{\Delta \nu }{f}}\overset{\text{HDV}}{\underset{\nu }{f}}-\overset{\text{HDV}}{\underset{\Delta x}{f}}}{{\left(\overset{\text{HDV}}{\underset{\Delta x}{f}}\right)}^{\text{2}}}=\frac{F_{\text{HDV}}}{{\left(\overset{\text{HDV}}{\underset{\Delta x}{f}}\right)}^{\text{2}}} \end{array}\right.\; \end{array}$$ (21)

#### 3.4.3 Long-wave limit equivalence and indispensability of frequency-domain analysis.

From the perspective of underlying physical mechanisms, the aforementioned algebraic discriminant in a weighted sum form is fundamentally equivalent to the Taylor expansion of the system’s frequency-domain transfer function as the perturbation frequency approaches zero (i.e., the long-wave ultra-low-frequency perturbation ω→0). To provide a rigorous theoretical proof, applying a second-order Taylor expansion to the magnitude $\left|G_{i}(j\omega )\right|$ of the single-vehicle frequency-domain response function derived in Section 3.2 at ω→0 yields:


$$\begin{array}{c} |G_{i}(j\omega )|\approx \text{1}-\left(\frac{\frac{\text{1}}{\text{2}}(\overset{i}{\underset{v}{f}}{)}^{\text{2}}-\overset{i}{\underset{v}{f}}\overset{i}{\underset{\Delta v}{f}}-\overset{i}{\underset{\Delta x}{f}}}{(\overset{i}{\underset{\Delta x}{f}}{)}^{\text{2}}\;}\right)\omega^{\text{2}}=\text{1}-G_{i}\omega^{\text{2}} \end{array}$$ (22)

Substituting this into the unified frequency-domain composite discriminant $\prod_{i}\;|G_{i}(j\omega ){|}^{P_{i}}\leq \text{1}$ constructed in Section 3.3, taking the natural logarithm of both sides, and utilizing the low-frequency infinitesimal limit approximation ln (1−x)≈−x, yields:


$$\begin{array}{c} \sum_{i}\;P_{i}\text{ln}\left(\text{1}-G_{i}\omega^{\text{2}}\right)\leq \text{0}⟹\sum_{i}\;P_{i}\left(-G_{i}\omega^{\text{2}}\right)\leq \text{0} \end{array}$$ (23)

Considering that the perturbation frequency ω>0, dividing both sides of the inequality by $-\omega^{\text{2}}$ and reversing the inequality sign ultimately yields:


$$\begin{array}{c} \sum_{i}\;P_{i}G_{i}\geq \text{0} \end{array}$$ (24)

This derivation shows that the algebraic discriminant is the long-wave, ultra-low-frequency limit of the frequency-domain criterion. Algebraic equations provide a low-frequency binary judgement, whereas the frequency-domain criterion retains the disturbance spectrum and can identify amplification hidden by the algebraic approximation when communication-degraded and heterogeneous states coexist.

## 4. Analytical stability and multidimensional heterogeneous impact assessment of mixed traffic flow

### 4.1 Topological characteristics of the stability domain and spatiotemporal dynamics consistency check in baseline scenarios

This section first evaluates a baseline mixed-flow scenario with CACC, ACC, and IDM from macroscopic stability-domain and time-domain perspectives.

#### 4.1.1 Topological evolution mechanism of the macroscopic stability domain in mixed traffic flow.

**Fig 2:**
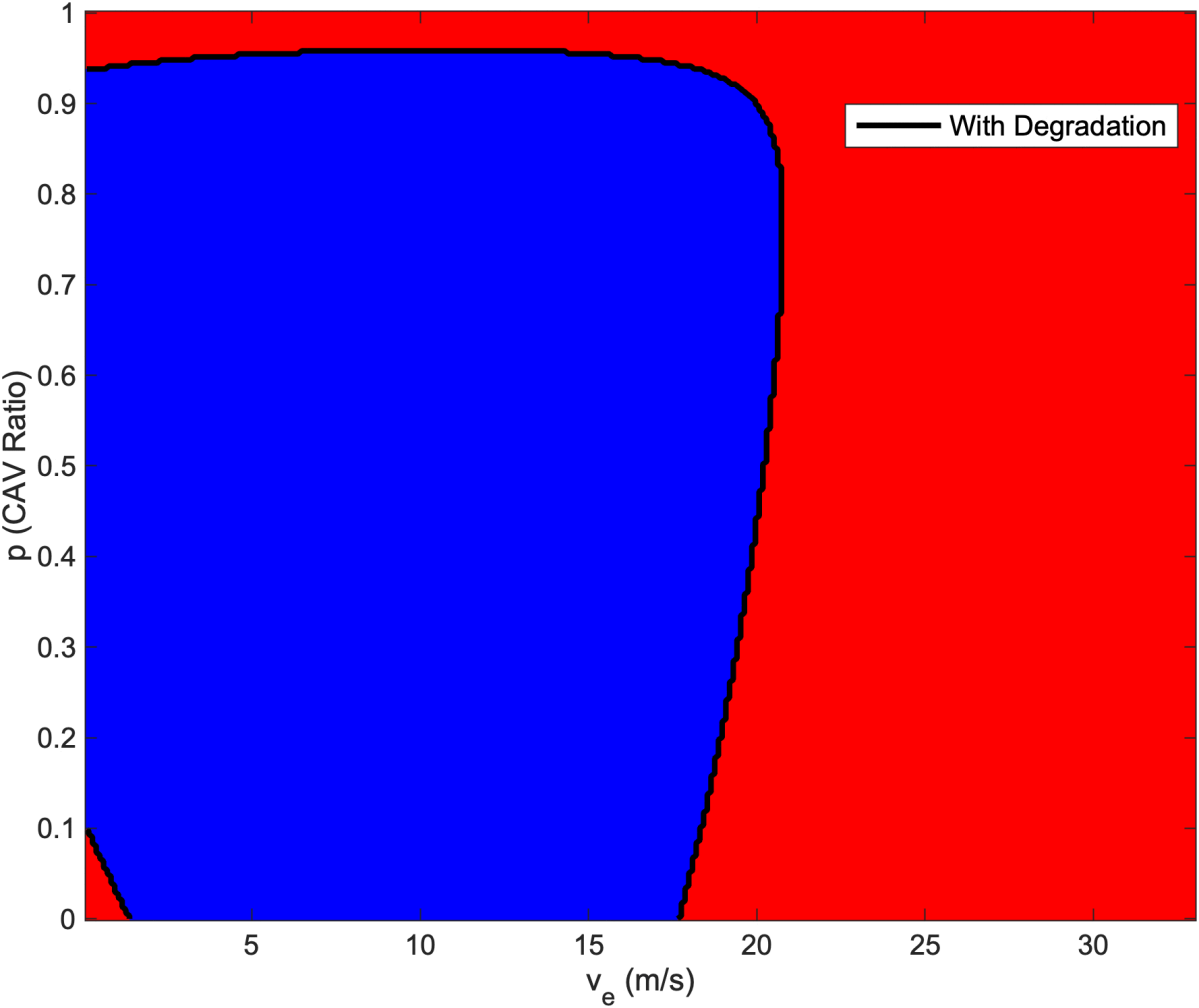
Macroscopic stability phase diagram of mixed traffic flow based on the unified frequency-domain criterion.

Figure 2 illustrates the macroscopic stability phase diagram, where the stability boundary satisfies $|G(j\omega ){|}_{max}=\text{1}$.

Figure 2 shows that stability changes across speed regimes. At $v_{e}>\text{2}\text{1}\text{m}/\text{s}$, larger spacing and time headways provide sufficient damping for stability across the examined penetration rates. At medium and low speeds, the boundary shifts nonlinearly with CAV penetration; for 0.2≤p≤0.8, the unstable region extends to about 21m/s. This is consistent with damping loss when CAVs following HDVs lose cooperative information and operate as degraded ACC vehicles. Full-speed stability returns only as p approaches 1.

#### 4.1.2 Spatiotemporal evolution consistency check of macroscopic theoretical boundaries.

To check the model-based consistency of these macroscopic boundaries, microscopic trajectory simulations of a 40-vehicle platoon at p=0.5 are conducted, applying a step deceleration perturbation to the leading vehicle at t≈10s.

**Fig 3:**
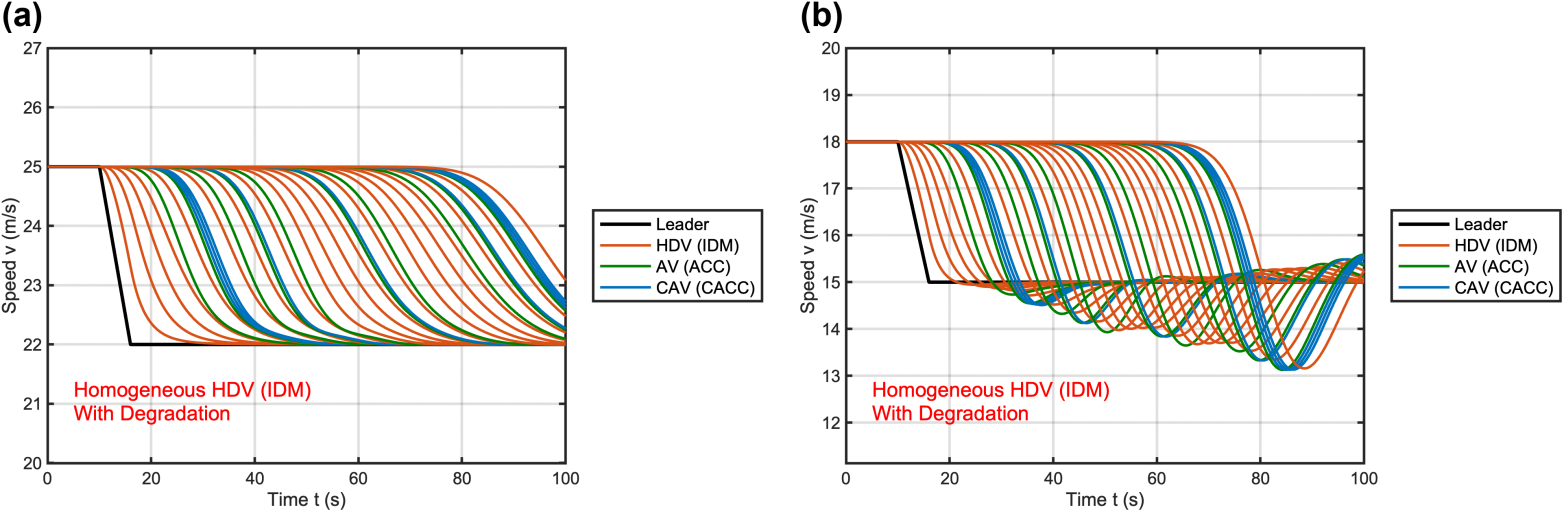
Model-based time-domain speed evolution comparison of the mixed platoon under step deceleration perturbation (𝐩=0.5).

As illustrated in Fig 3(a), at an equilibrium speed $v_{e}=\text{2}\text{5}\text{m}/\text{s}$ (decelerating to 23m/s), the system exhibits robust low-pass filtering. The perturbation amplitude monotonically decreases upstream without nonlinear distortion, achieving asymptotic convergence and remaining consistent with the stable region predicted by the frequency-domain criterion.

In Fig 3(b), when $v_{e}=\text{1}\text{8}\text{m}/\text{s}$ (decelerating to 15 m/s), the system enters the unstable region. The perturbation amplifies upstream because local CAV cooperation cannot absorb HDV braking, causing the tail speed to fall below 13.5 m/s and producing a stop-and-go wave. This comparison is consistent with the frequency-domain boundary and provides a baseline for heterogeneity analyses.

### 4.2 Comparison of macroscopic stability boundaries between algebraic and frequency-domain criteria

**Fig 4:**
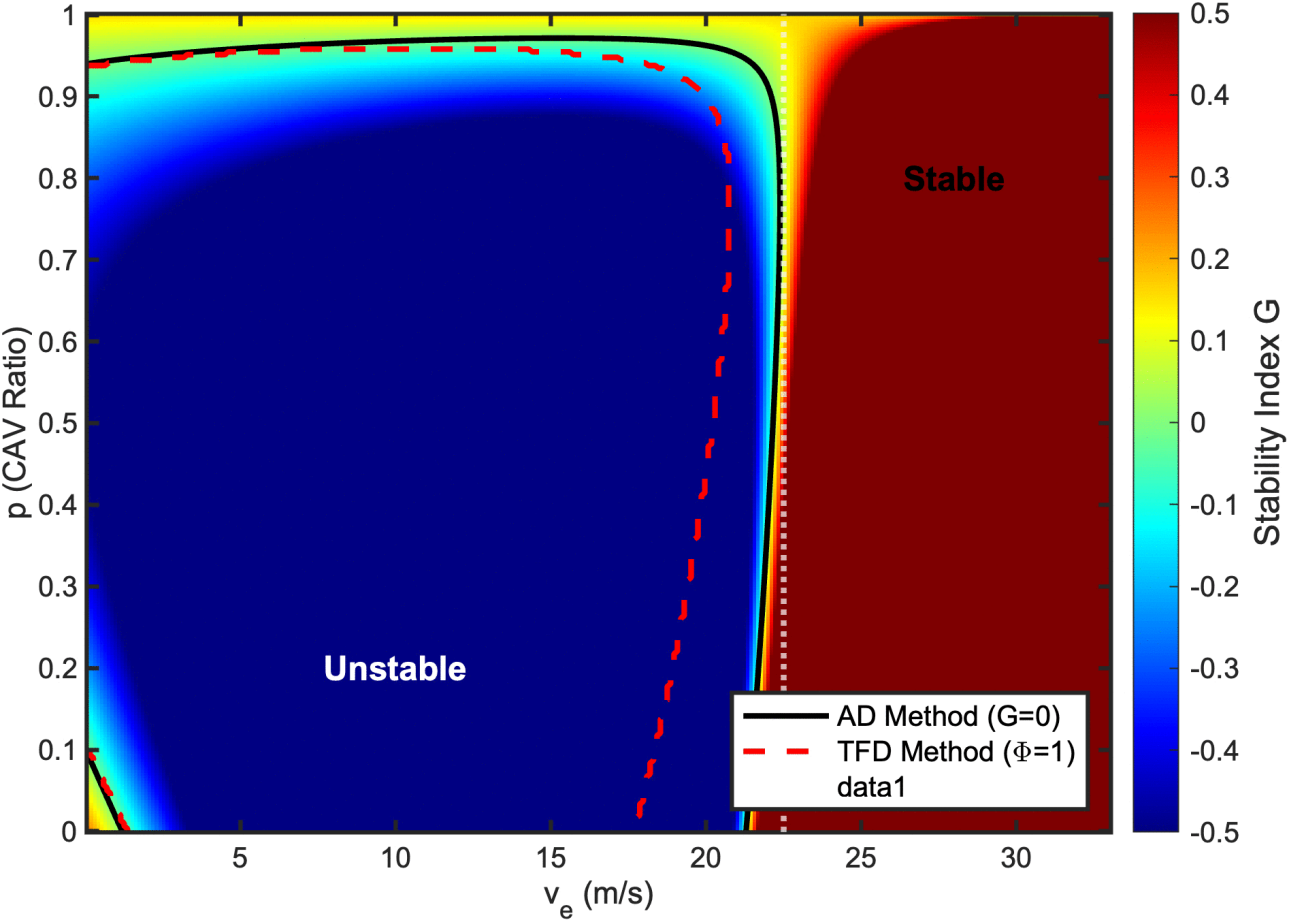
Comparison phase diagram of the stability boundaries under the algebraic and frequency-domain discriminants for mixed traffic flow.

To verify theoretical equivalence and assess evaluation bias, Fig 4 compares the algebraic stability index heatmap G with the frequency-domain topological boundary max|G(jω)|=1.

Figure 4 shows that algebraic and frequency-domain boundaries overlap near p = 0 or p = 1, where homogeneous or near-homogeneous cases are dominated by low-frequency behavior. For 0.1≤p≤0.9, CACC, degraded ACC, and HDV states coexist and a clear mismatch appears: the algebraic boundary shifts toward higher speeds and classifies part of the frequency-domain stable region as unstable. This reflects the limitation of averaging distinct microscopic response mechanisms into a low-frequency algebraic index.

### 4.3 Penalty effects of the CAV communication degradation mechanism and boundary evolution of an ideal connected-upgrade benchmark

During the transition toward connected automation, HDVs cannot provide cooperative V2X information to following CAVs. In the baseline degradation mechanism, those CAVs lose preceding-vehicle state input and passively operate as ACC vehicles, reducing local damping. This section compares the degraded boundary with an ideal connected-upgrade benchmark to quantify the potential stability benefit of maintaining cooperative information.

#### 4.3.1 Macroscopic stability domain evolution under communication degradation and the ideal connected-upgrade benchmark.

**Fig 5:**
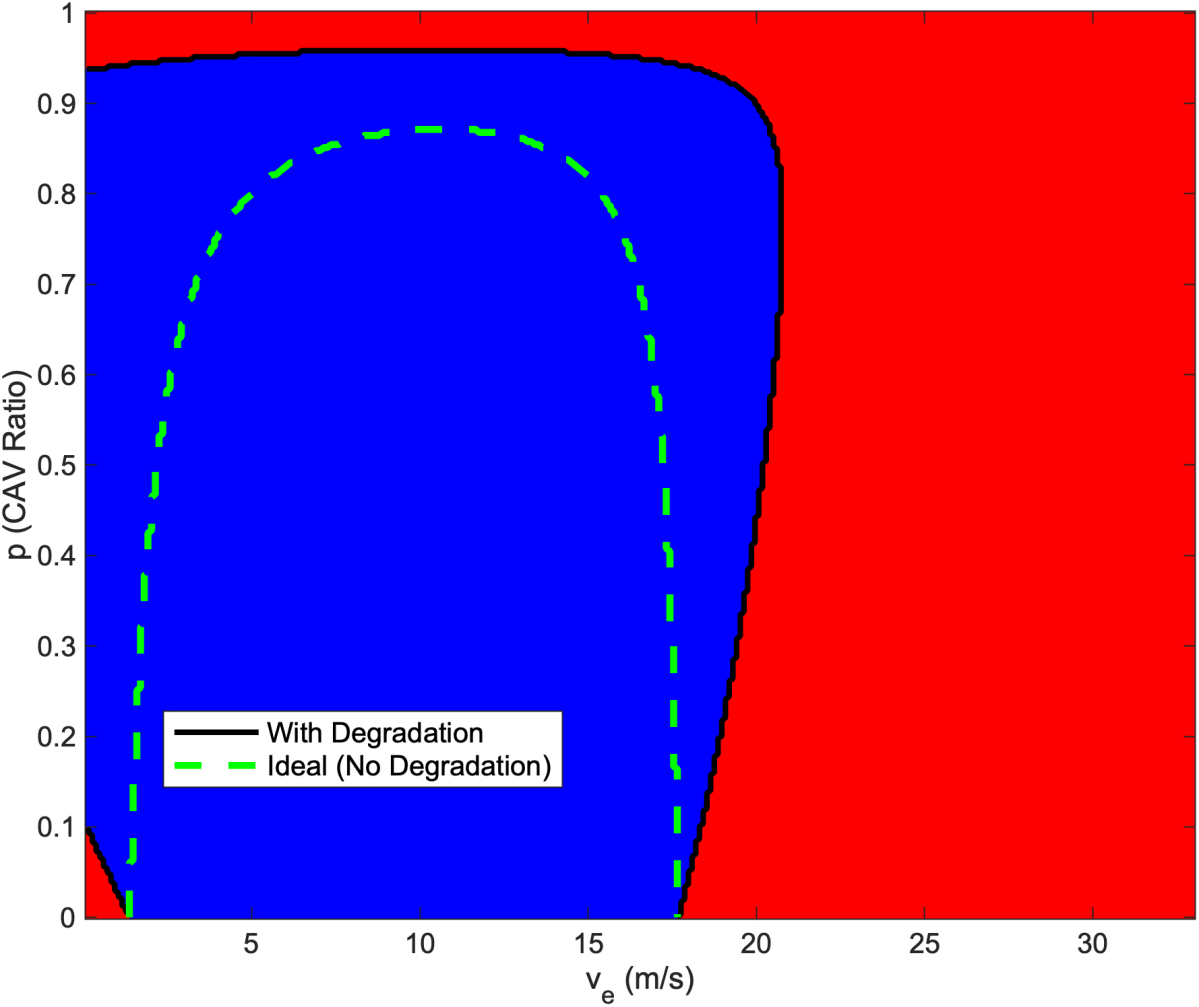
Comparison phase diagram of macroscopic stability boundaries for mixed traffic flow under communication degradation and the ideal connected-upgrade benchmark.

Figure 5 illustrates the macroscopic stability phase diagram, comparing the degraded mixed-flow boundary with the ideal connected-upgrade benchmark boundary.

The enclosed region explicitly delineates the extent to which communication degradation weakens the disturbance rejection capability of the mixed traffic flow. At a moderate CAV penetration rate of p=0.7, the ideal connected-upgrade benchmark constrains the critical instability speed to approximately $v_{e}=\text{1}\text{7}\text{m}/\text{s}$. However, under the degradation mechanism, this boundary shifts significantly toward the high-speed regime, extending the critical speed to $v_{e}=\text{2}\text{0}.\text{5}\text{m}/\text{s}$ and signifying a substantial reduction in low-frequency perturbation dissipation capacity during medium- and high-speed free-flow phases.

For the global stability threshold, the ideal connected-upgrade benchmark achieves string stability across all speeds at approximately p≈0.873. By contrast, the degraded baseline requires approximately p≈0.960. This threshold difference suggests that cooperative information availability can substantially improve the modelled stability margin, although this benchmark should not be interpreted as direct validation of a specific deployment strategy.

#### 4.3.2 Time-domain consistency check of degradation and connected-upgrade effects.

To examine the model-based consistency of this boundary misalignment, microscopic trajectory simulations of a 40-vehicle platoon are conducted at a critical coordinate ($p=\text{0}.\text{7},v_{e}=\text{2}\text{0}\text{m}/\text{s}$), applying a step deceleration to the leading vehicle to establish a new equilibrium of 17m/s.

**Fig 6:**
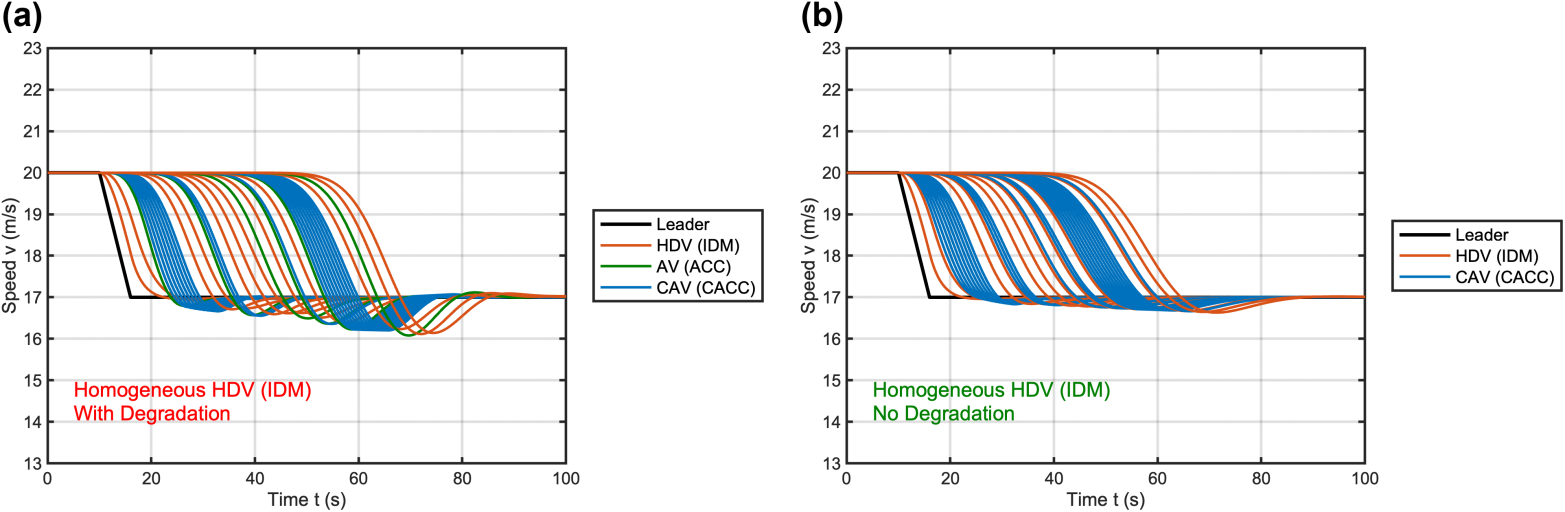
Model-based time-domain speed evolution comparison of the mixed platoon under step deceleration perturbation (𝐩=0.7).

Figure 6(a) shows that passive ACC degradation weakens local damping: the perturbation amplifies upstream, tail vehicles lag, and speed undershoots reach 15.8 m/s. In the ideal connected-upgrade benchmark (Fig 6b), cooperative information remains available, CAVs retain CACC mode, and the platoon converges smoothly. These simulations are consistent with Fig 5 and support the potential disturbance-rejection benefit of maintaining cooperative information.

### 4.4 Penalty effects and microscopic evolution of HDV multidimensional heterogeneity

This section represents HDV heterogeneity with an equal mixture of IDM, OVM, and RSM. IDM captures safety-distance and relative-speed-coupled response, OVM captures spacing-driven response without explicit relative-speed feedback, and RSM captures risk-sensitive stochastic response.

#### 4.4.1 Topological evolution of macroscopic stability domains under multidimensional heterogeneous impacts.

**Fig 7:**
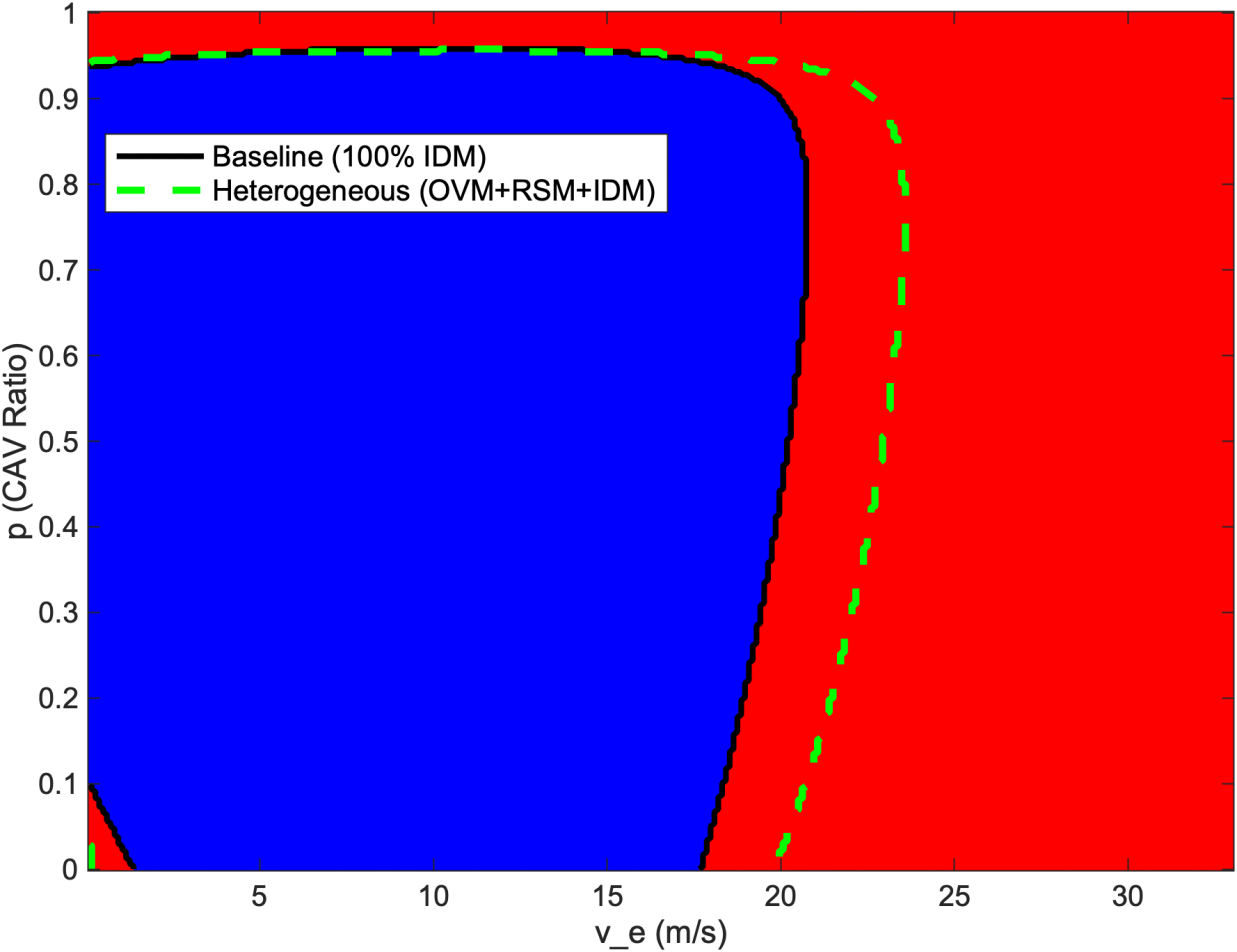
Comparison of macroscopic stability boundaries for mixed traffic flow under HDV multidimensional heterogeneity.

Figure 7 compares the macroscopic boundaries of an IDM-only baseline with the dual heterogeneity framework, revealing two distinct topological impacts on the stability domain.

As p approaches 1, IDM-only and heterogeneous-HDV cases have similar global thresholds (about p≈0.96), indicating little change in the full-speed stability threshold. However, HDV heterogeneity compresses the local stable region at 15–25 m/s; at p=0.4, the critical boundary shifts from 18 to about 23 m/s. Thus, mechanical hysteresis and risk-sensitive fluctuations reduce local disturbance rejection even when the global threshold is similar.

#### 4.4.2 Spatiotemporal dynamics consistency check of dual heterogeneity coupling.

To examine this microscopic evolution, a critical coordinate within the misalignment region with a penetration rate of p=0.6 and an initial equilibrium speed of 23 m/s is tested, with the leader decelerating to 20 m/s at t=15s.

**Fig 8:**
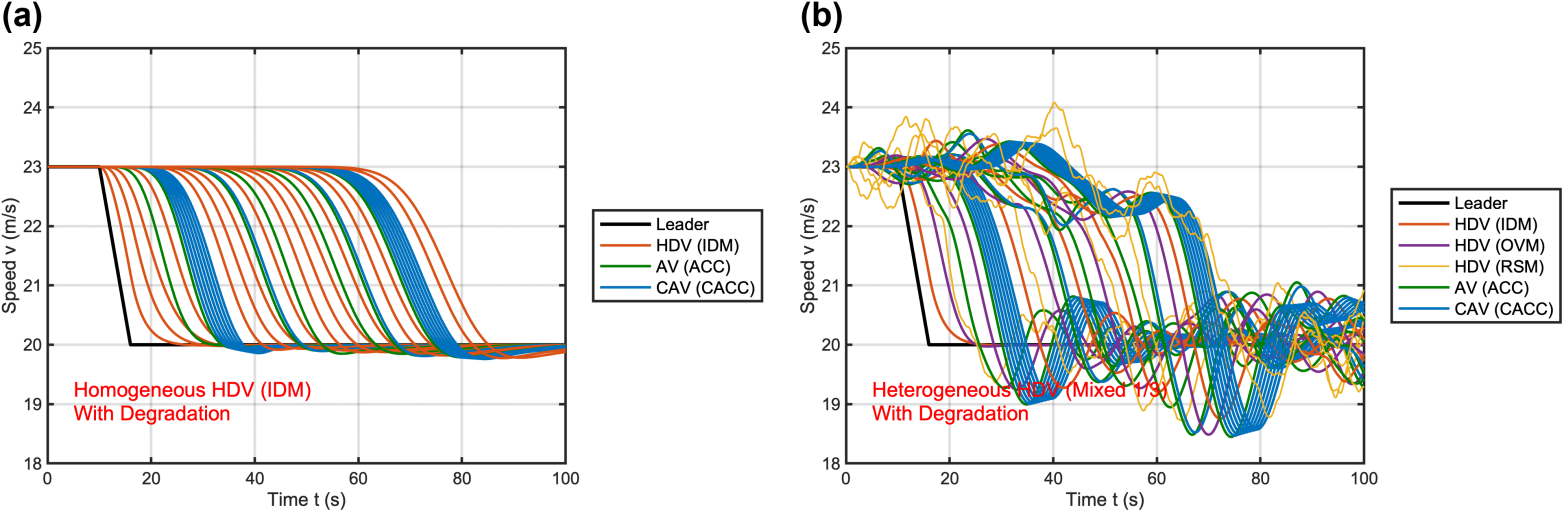
Comparison of microscopic spatiotemporal dynamics evolution of the mixed platoon in the critical phase (*p* = 0.6).

Figure 8 compares homogeneous and heterogeneous-HDV dynamics. The homogeneous baseline dissipates the perturbation, consistent with stable low-pass filtering. In the heterogeneous case, HDV responses generate high-frequency fluctuations before the imposed perturbation; as the deceleration wave propagates, the flexible-response component brakes more strongly and the local speed trough reaches 18.5 m/s. The imposed wave interacts with internal fluctuations and develops into a backward-propagating stop-and-go wave, indicating weaker local damping in critical regimes.

The following subsections test four robustness and scope issues: HDV parameter uncertainty, functional cooperative-information reliability loss, equivalent response delay, and US101 replay of the IDM-based HDV component. These analyses test robustness and define the empirical boundary of the model-based framework.

### 4.5 Robustness under HDV parameter uncertainty

To test dependence on a single deterministic parameter set, N=100  Latin Hypercube samples perturb selected HDV parameters by ±10% around nominal values. The sampled parameters are IDM A, B, $s_{\text{0}}$, $\tau_{h}$, V; OVM κ, λ, d, and $v_{\text{0}}$; and RSM $w_{c}$, τ, and $\sigma_{a}$. CACC and ACC controller parameters remain fixed to isolate HDV-side uncertainty.

**Fig 9:**
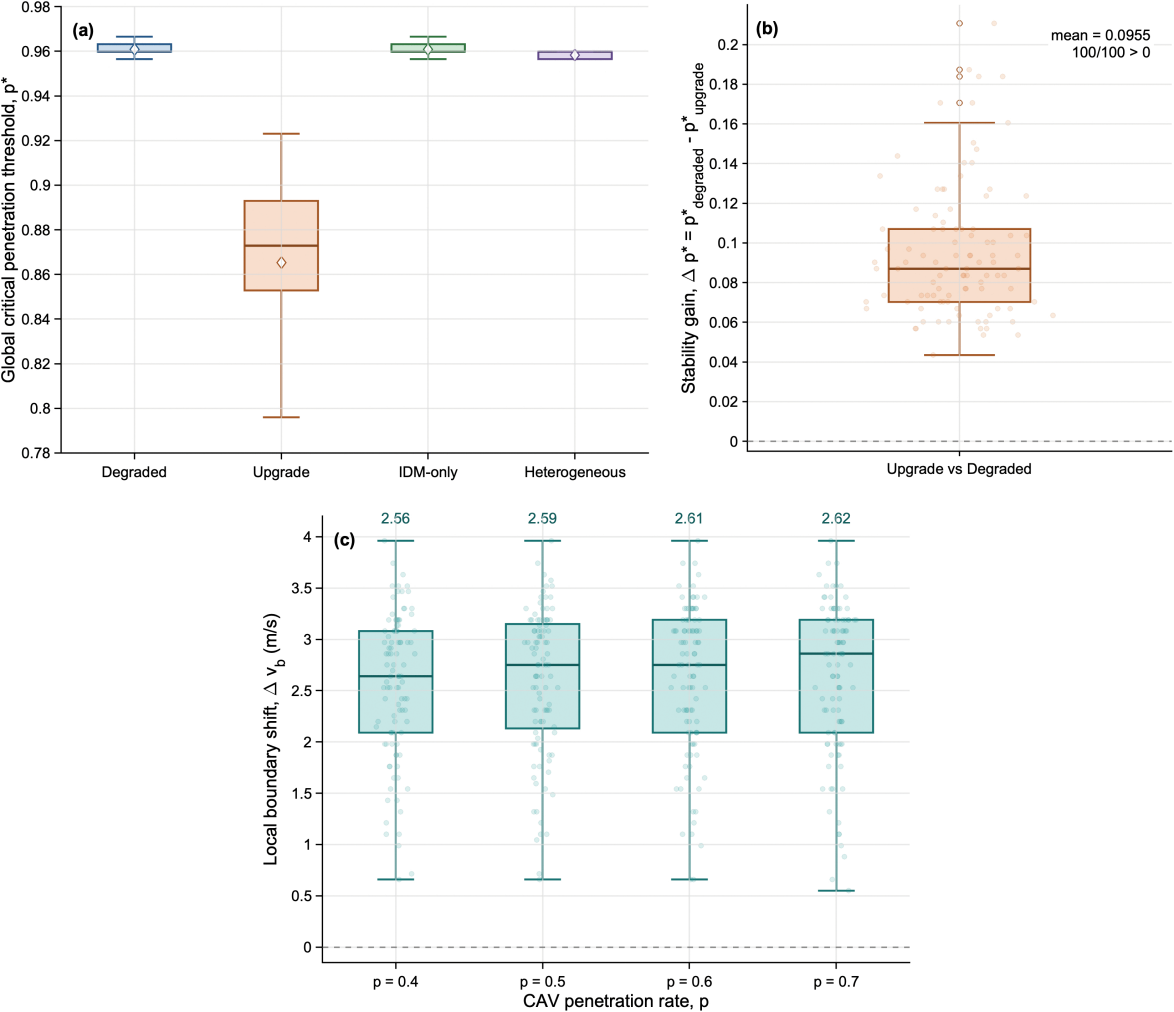
Robustness of critical stability thresholds and local boundary shifts under HDV parameter uncertainty.

Figure 9(a) shows the global critical threshold distribution. For the degraded baseline, the nominal $p^{*}$ is 0.9599 and the uncertainty samples give $p^{*}=\text{0}.\text{9608}\pm \text{0}.\text{0034}$; the 5%, 50%, and 95% quantiles are 0.9564, 0.9599, and 0.9666. Thus, the high critical penetration requirement is not driven by one HDV parameter set.

The connected-upgrade benchmark remains beneficial: $\Delta p^{*}=\overset{*}{\underset{\text{degraded}}{p}}-\overset{*}{\underset{\text{upgrade}}{p}}$ is positive in all 100 samples, with a mean reduction of 0.0955.

Figure 9(c) shows that HDV structural heterogeneity mainly affects local boundaries. Although IDM-only and heterogeneous-HDV global $p^{*}$ values remain close, local boundary-speed shifts are positive at p = 0.4, 0.5, 0.6, and 0.7, with mean shifts of 2.5588, 2.5896, 2.6078, and 2.6188 m/s. HDV heterogeneity therefore compresses local stable-speed regions more than it changes the global threshold.

Overall, parameter uncertainty supports the main conclusions: degradation imposes a high global threshold, the connected-upgrade benchmark reduces it, and HDV heterogeneity mainly reshapes local boundaries.

### 4.6 Sensitivity to functional communication reliability loss

To examine communication degradation beyond topology, a reliability-loss parameter ρ is introduced. In the baseline, a CAV degenerates from CACC to ACC when following an HDV. Here, ρ denotes the probability that cooperative information is unavailable even when a CAV follows another CAV; it is a functional reliability parameter, not a packet-loss model.

With this extension, the probability weights are modified as $P_{\text{CACC}}=p^{\text{2}}(\text{1}-\rho ),{\;P}_{\text{ACC}}=p(\text{1}-p)+p^{\text{2}}\rho ,$ $P_{\text{HDV}}=\text{1}-p.$

**Fig 10:**
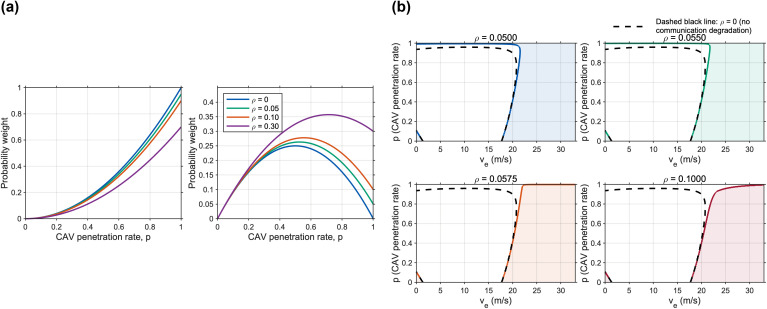
Effects of functional communication reliability loss on probability weights and stability domains.

As shown in Fig 10(a), increasing ρ reduces the CACC weight and increases the degraded ACC weight. Part of the cooperative damping contribution is therefore transferred to the degraded car-following mode, weakening the disturbance-rejection capability of the mixed traffic flow.

Figure 10(b) shows that increasing ρ compresses the stable domain. At ρ=0.0500, the stable region shifts toward high penetration; at ρ=0.0550, full-speed stability is marginal near p = 1; at ρ=0.0575, no global full-speed threshold is detected within p∈[0,1], although local stable regions remain; at ρ=0.1000, the stable region further shrinks. Functional reliability loss therefore changes both the critical requirement and domain shape.

This contraction also appears in $p^{*}$: the baseline gives $p^{*}=\text{0}.\text{959866}$, whereas ρ=0.0500 gives $p^{*}=\text{0}.\text{996656}$. The refined scan indicates a transition between ρ=0.0500 and ρ=0.0575, beyond which no full-speed threshold appears within p∈[0,1].

### 4.7 Sensitivity to equivalent response delay

An equivalent delay τd is added to represent perception, communication, computation, and actuation lag. The delay-free case ($\left(\tau_{d}=\text{0}\right)$) is the baseline; $\tau_{d}=\text{0}.\text{05}$, 0.10, and 0.20s test mild-to-moderate response lag before the adopted CACC unit develops intrinsic delay-induced amplification.

Unlike the reliability-loss analysis, this delay-sensitivity test does not modify the state probabilities; the degraded-baseline weights remain $P_{\text{CACC}}=p^{\text{2}},{\;P}_{\text{ACC}}=p(\text{1}-p),$ $P_{\text{HDV}}=\text{1}-p$. Instead, each delay-free transfer function is replacedby $G_{n}(s,\tau_{d})=\frac{e^{-s\tau_{d}}(\overset{n}{\underset{\Delta v}{f}}s+\overset{n}{\underset{h}{f}})}{s^{\text{2}}+e^{-s\tau_{d}}\left[(\overset{n}{\underset{\Delta v}{f}}-\overset{n}{\underset{v}{f}})s+\overset{n}{\underset{h}{f}}\right]}$, with s=jω, in the same probability-weighted stability criterion.

**Fig 11:**
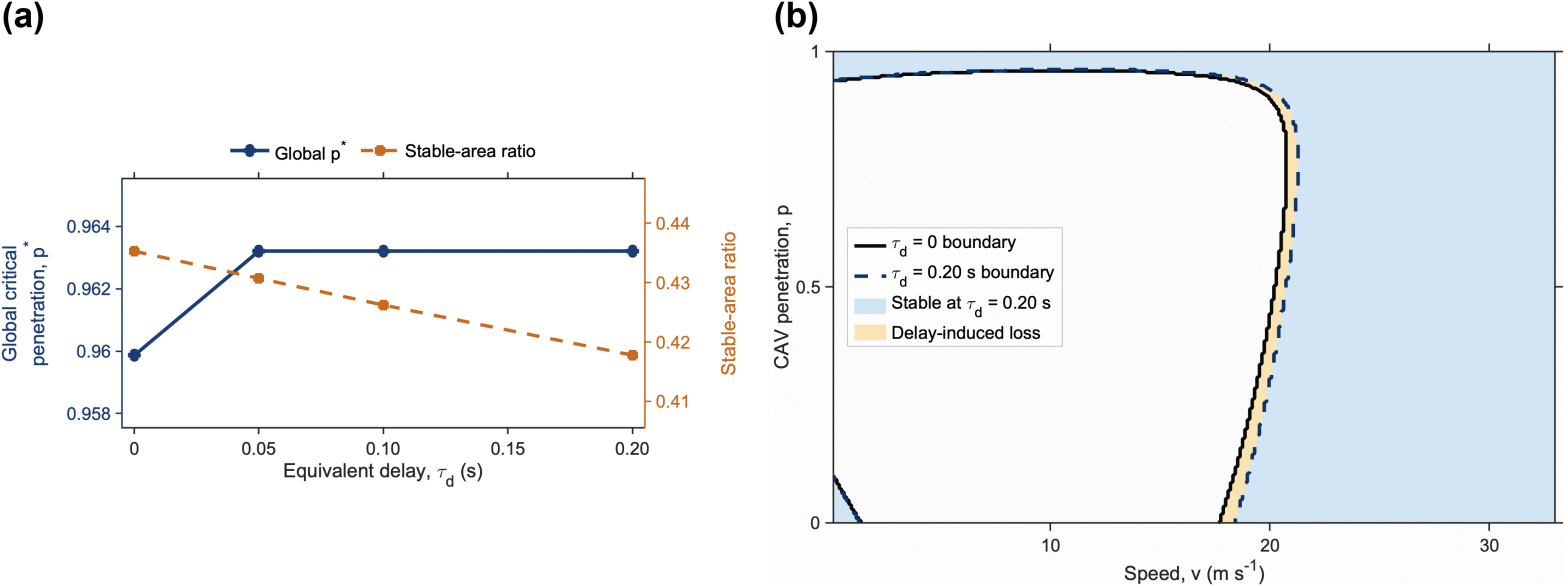
Effects of equivalent response delay on stability metrics and stability-domain shrinkage in mixed traffic flow.

Figure 11(a) shows that τd only slightly affects the global threshold, from $p^{*}=\text{0}.\text{9599}$ at $\tau_{d}=\text{0}$ to about $p^{*}=\text{0}.\text{9632}$ at $\tau_{d}=\text{0}.\text{20}\;\text{s}$. However, the stable-area ratio decreases continuously, indicating loss of stability margin across the v-p plane.

Figure 11(b) compares the delay-free and $\tau_{d}=\text{0}.\text{20}\;\text{s}$ domains. The delayed case visibly shrinks the stable region while leaving the global threshold close to baseline, so equivalent delay mainly reduces stability-domain extent rather than sharply shifting $p^{*}$.

### 4.8 Empirical time-domain replay of the IDM-based HDV component using US101 trajectories

To provide trajectory-based evidence for the HDV component, IDM replay is conducted using NGSIM US101 car-following data. Continuous passenger-car following segments require stable leader-follower relationships, unchanged follower lanes, speeds of 5−30 m/s, net spacings of 5−120 m, and duration of at least 12 s. Leader-deceleration events require acceleration below −0.3 m/s² for at least 1 s and cumulative speed drop of at least 1.0 m/s within 5 s, identifying mild deceleration rather than hard braking.

For each event, the observed leader trajectory drives the IDM follower model, and simulated follower trajectories are compared with observations. IDM parameters match the frequency-domain analysis and are not event-recalibrated. A total of 266 valid events remain, with no invalid or collision replay cases.

**Fig 12:**
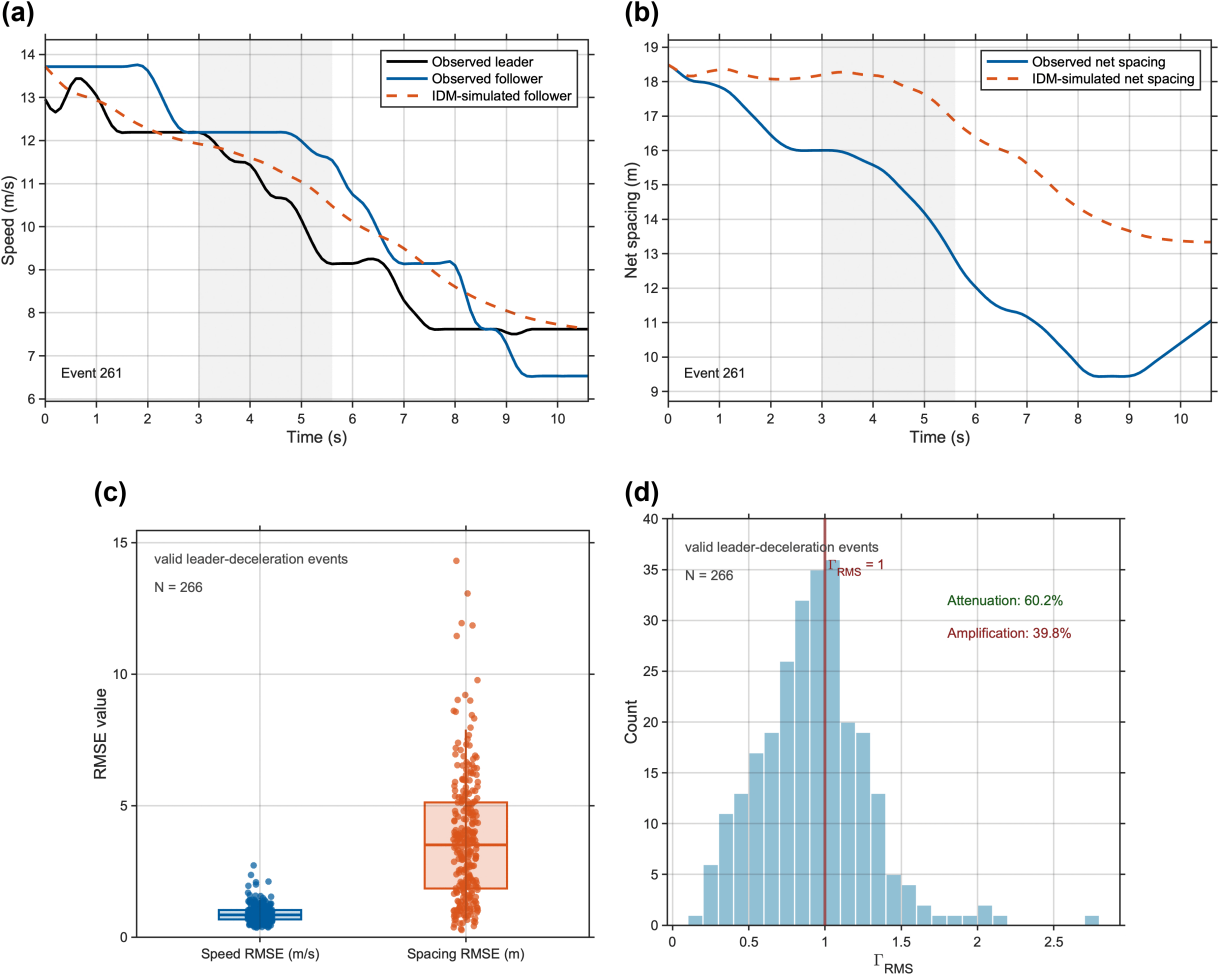
US101-based IDM trajectory replay and disturbance-response characteristics of HDV car-following events.

Figure 12(a), 12(b) show one representative replay. The IDM follower captures the main observed speed-reduction trend and the overall spacing-decrease process. Across valid events, median speed RMSE is 0.8456 m/s and median spacing RMSE is 3.5078 m; speed is reproduced more accurately than spacing, but simulated spacing follows the observed decreasing trend.

Figure 12(d) gives median ΓRMS = 0.9186; 60.2% of events attenuate disturbances $\left(\Gamma_{\text{RMS}}<\text{1}\right)$, whereas 39.8% amplify them $\left(\Gamma_{\text{RMS}}>\text{1}\right)$. Thus, real HDV responses show both attenuation and amplification. The US101 replay supports the IDM-based HDV component, while the full v-p stability boundary remains a model-based frequency-domain result.

## 5. Conclusions and future work

This study examined how cooperative-information loss and heterogeneous HDV responses affect string stability during the transition toward connected automation. It developed a probability-weighted full-frequency transfer-function framework combining CAV, degraded AV, and HDV. The contribution is not new car-following models, but the integration of communication-state degradation and HDV heterogeneity into a frequency-domain criterion that preserves vehicle-specific disturbance responses.

(1) The analysis clarifies that the algebraic condition is the low-frequency, long-wave limit of the frequency-domain formulation. It can miss boundary changes at intermediate penetration rates where CAV, degraded AV, and HDV coexist. Under the adopted linearised small-perturbation setting, the full-frequency criterion gives a more complete model-based stability description than the algebraic approximation alone.
(2) Communication degradation imposes a substantial stability penalty. In the degraded baseline, CAVs following HDVs lose cooperative information and operate as ACC vehicles, weakening damping. The nominal full-speed threshold is about 0.960, compared with about 0.873 for the ideal connected-upgrade benchmark. Reliability-loss analysis further shows stable-domain compression and a threshold of about 0.997 at 5% reliability loss. These results support the importance of cooperative information availability, while the benchmark remains an ideal model case rather than deployment evidence.
(3) HDV heterogeneity mainly reshapes local stability domains. Compared with IDM-only traffic, heterogeneous HDV responses reduce stability margins in transitional speed regimes. The ± 10% HDV-side parameter uncertainty analysis supports this interpretation: the degraded threshold remains narrow (mean 0.9608, SD 0.0034), and the connected-upgrade benchmark reduces the threshold in all samples. Thus, local boundary location is parameter-sensitive, but the main conclusion is robust.
(4) Delay and trajectory-replay analyses clarify the modeling boundary. Equivalent delay reduces stable-area ratio even when p* changes only slightly at 0.20 s, so the delay-free case is an optimistic stability reference. US101 replay supports the IDM-based HDV component across 266 events, with median speed and spacing RMSEs of 0.8456 m/s and 3.5078 m and both attenuation and amplification observed. Because US101 lacks CAV/ AV and communication-state labels, it does not validate the full speed-penetration map.

Overall, mixed-flow stability depends on CAV penetration, cooperative-information availability, and HDV frequency-response heterogeneity. Practical measures such as V2X deployment, variable speed limits, or dedicated CAV lanes should therefore be treated as future hypotheses for controller-, communication-, and traffic-flow-level evaluation, not conclusions established here.

Limitations remain. The framework is restricted to single-lane longitudinal car-following and excludes lane changing, merging, diverging, and dedicated platoon formation. Communication degradation is represented by passive CACC-to-ACC transition and a functional reliability-loss parameter, not packet-level V2X connectivity with vehicle-specific delays. US101 replay supports only the IDM-based HDV component; OVM and RSM require further calibration with large trajectory datasets. Future work should include lateral interactions, organized platooning, packet-level communication reliability, heterogeneous delays, and datasets or experiments with CAV/ACC/CACC states and communication-quality labels.
